# A case of tumor-like inflammatory demyelinating disease with progressive brain and spinal cord involvement: Um caso de doença desmielinizante inflamatória semelhante a tumor com envolvimento progressivo de cérebro e medula espinhal

**DOI:** 10.1590/1516-3180.2014.7832407

**Published:** 2014-11-07

**Authors:** Xu Zhi Peng, Li Hong Hua, Sun Zhi Qiang, Wu Qiang

**Affiliations:** I MD, MSc. Attending Physician, Department of Neurology, Wuhan General Hospital of Guangzhou Command, Wuhan, China; II MD, PhD. Professor, Department of Neurology, Wuhan General Hospital of Guangzhou Command, Wuhan, China; III MD, MSc. Attending Physician, Department of Radiology, Wuhan General Hospital of Guangzhou Command, Wuhan, China

**Keywords:** Demyelinating diseases, Brain, Spinal cord, Radiology, Pathology, Doenças desmielinizantes, Cérebro, Medula espinhal, Radiologia, Patologia

## Abstract

**CONTEXT:**

Tumor-like inflammatory demyelinating disease (TIDD) usually occurs in the brain and rarely occurs in the spinal cord. TIDD appears to be very similar to tumors such as gliomas on imaging, which may lead to incorrect or delayed diagnosis and treatment.

**CASE REPORT:**

Because of headache and incoherent speech, a 24-year-old Chinese male presented to our hospital with a two-week history of respiratory infections. After dexamethasone treatment, his symptoms still got worse and surgery was performed for diagnostic purposes. Histological examination revealed that the lesion was inflammatory. Further lesions appeared in the spine (T3 and T4 levels) after two months and in the right occipital lobe after three months. After intravenous immunoglobulin (IVIG) and methylprednisolone treatment, his symptoms improved.

**CONCLUSION:**

Progressive lesions may damage the brain and spinal cord, and long-term prednisolone and IVIG therapy are beneficial in TIDD patients.

## Abstract (pt)

**CONTEXTO:**

A doença desmielinizante inflamatória tumoral (DDIT) geralmente ocorre no cérebro e raramente na medula espinhal. A DDIT é muito semelhante a tumores tais como gliomas em exames de imagem, o que pode conduzir a diagnóstico e tratamento tardios e incorretos.

**RELATO DO CASO:**

Por causa de dor de cabeça e discurso incoerente, um homem chinês de 24 anos de idade foi ao hospital com história de duas semanas de infecções respiratórias. Após o tratamento com dexametasona, seus sintomas ficaram ainda piores e a cirurgia foi realizada para fins de diagnóstico. O exame histológico revelou que a lesão era inflamatória. Mais lesões apareceram na coluna vertebral (níveis T3 e T4) após dois meses, e no lobo occipital direito depois de três meses. Depois de tratamento com imunoglobulina intravenosa (IGIV) e metilprednisolona, seus sintomas melhoraram.

**CONCLUSÃO:**

Lesões progressivas podem danificar o cérebro e a medula espinhal, e prednisolona a longo prazo e terapia de IGIV são benéficas em pacientes DDIT.

## INTRODUCTION

Tumor-like inflammatory demyelinating disease (TIDD) is a rare central nervous system (CNS) demyelinating disorder affecting the cerebral hemispheres or the spinal cord.^1^ TIDD can be difficult to diagnose because the inflammatory lesion clinically and radiologically mimics a tumor in the early stages.^2^ Its exact incidence and etiology remain unknown.^3^ Despite its rarity, awareness of this entity is important for guiding clinical practice, since a delayed diagnosis or misdiagnosis may deny patients the full benefit of a complete recovery or result in unnecessary aggressive management, thereby having a deleterious effect.^4^

TIDD usually occurs in the brain and rarely occurs in the spinal cord. TIDD with both brain and spinal cord involvement is even rarer, with only two reported cases.^1^,^5^ Because of the presence of the demyelinating mechanism, corticosteroids may provide effective treatment. Only a small percentage of TIDD patients do not respond to corticosteroid therapy. The following case report is an example of TIDD that was diagnosed in our hospital. Because the patient was not corticosteroid-sensitive, progressive lesions occurred in his brain and spinal cord. The report focuses on the difficulties in diagnosis and treatment, and also presents a review of the literature.

## CASE REPORT

A 24-year-old right-handed male was admitted to the Neurology Department of Wuhan General Hospital of Guangzhou Command, complaining of headache and incoherent speech for two days.

The patient had reported a history of cold with coughing and fever about two weeks earlier. He had been treated with anti-cold medicine for several days and these symptoms had resolved one week prior to the admission of this report. He was a college student, and unmarried. He did not smoke and did not use alcohol. Prior to these occurrences, he had been in good health, with no relevant past medical or family history. He had received routine immunizations at an early age without any side effects. He did not have any history of travel, exposure to animals or contact with sick people over recent months.

On arrival at our center, he reported experiencing severe headache. The physical examination was unremarkable, with normal blood pressure of 120/80 mmHg, heart rate of 85 bpm, respiratory rate of 18/min and temperature of 36.8 °C. Neurological examination revealed cognitive impairment, neck rigidity, positive Kernig's sign and negative upper and lower Brudzinski's signs. All of the following blood test parameters were within the reference range, namely: white blood cell and platelet counts, liver function tests, renal function, electrolytes, C-reactive protein and erythrocyte sedimentation rate. Autoimmune antibody and serological screening for HIV, syphilis and hepatitis B and C were all negative.

**Figure 1:**
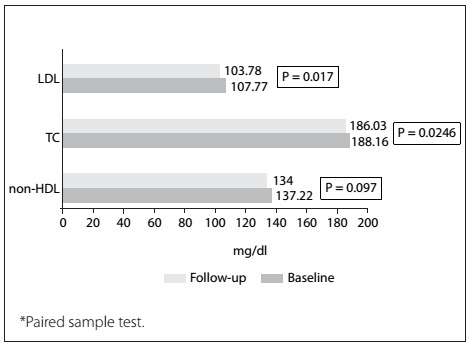
Magnetic resonance imaging (MRI) of brain on admission, showing: hypointense image with T1 weighting (A); hyperintense image with T2 weighting (B); enhancement after gadolinium-diethylenetriamine pentaacetic acid (Gd-DTPA) administration, with T1 weighting (C); and magnetic resonance spectroscopy (^1^H-MRS) with increased choline (Cho) and decreased N-acetylaspartate (NAA) peaks (D).

A lumbar puncture was subsequently performed, and analysis of the cerebrospinal fluid (CSF) revealed elevated opening pressure (250 mmH_2_O; normal < 180 mmH_2_O) and elevated total protein (51 mg/dl; normal < 40 mg/dl). White blood cell and red blood cell counts and glucose and chloride levels were normal in the CSF. Gram staining and bacterial, fungal and mycobacterial cultures on the CSF were negative. The immunoglobulin G (IgG) index was normal without oligoclonal bands. An electroencephalogram (EEG) showed that diffuse intermittent slow waves appeared predominantly in the left temporal area. Visual evoked potential (VEP) and brainstem auditory evoked potential (BAEP) were also within normal limits. Magnetic resonance imaging (MRI) on the brain revealed an irregular well-defined mass measuring 5 cm in the left basal ganglia, with surrounding brain edema. The lesion was hypointense on the T1-weighted images and hyperintense on the T2-weighted images, with heterogeneous enhancement after gadolinium-diethylenetriamine pentaacetic acid (Gd-DTPA) administration (**Figure 1**). The initial spinal cord MRI was normal. Proton magnetic resonance spectroscopy (^1^H-MRS) provides insight into the chemical composition of lesions and it revealed an elevated choline (Cho) peak and suppressed N-acetylaspartate (NAA) peak, although the brain spectrum was noisy (**Figure 1**). In view of the case history and complementary examinations, the diagnosis of a demyelinating lesion was considered. Therefore, he was initially treated with dexamethasone (intravenous, IV, 10 mg/day) and acyclovir (IV, 500 mg/8 hours) for one week. Unfortunately, his symptoms of headache and cognitive impairment became markedly worse.

**Figure 2:**
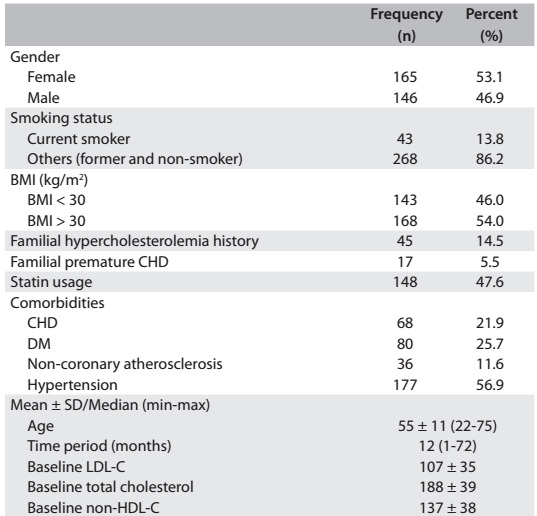
Pathological examination showing that the lesion consisted of dense accumulation of macrophages, lymphocytes and reactive astrocyte (A-B).

The differential diagnosis included brain demyelinating disease and brain tumor. Because of non-response to corticosteroid therapy and contrast enhancement on MRI, a low-grade glioma was suspected and surgery was carried out to clarify the diagnosis. Histopathological analysis on the biopsied tissue showed moderate perivascular accumulation of small lymphocytes and a large number of macrophages with reactive astrocytes (**Figure 2**). On imaging, the lesion appeared to be very similar to intracranial tumors such as gliomas. From the histopathology of the lesion, a diagnosis of tumor-like inflammatory demyelinating disease of the brain was made.

**Figure 3:**
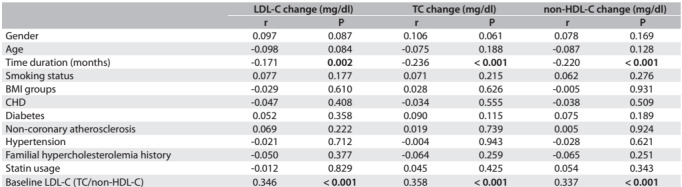
Magnetic resonance imaging of brain, four weeks after admission, showing: hyperintense image with T2 weighting (A); and enhancement after gadolinium-diethylenetriamine pentaacetic acid (Gd-DTPA) administration (B).

The patient was treated with oral dexamethasone (0.75 mg, three times a day) continuously, but his condition continued to deteriorate. Four weeks later, he suddenly developed right hemiparesis and had difficulty in walking. On neurological examination, he presented upper motor neuron weakness grade 4/5 in the right upper and lower limb. A follow-up MRI on the brain demonstrated an increase in the mass effect and edema in the left basal ganglia region. The large mass lesion was still hypointense on the T1-weighted images and hyperintense on the T2-weighted images, with heterogeneous enhancement after Gd-DTPA administration (**Figure 3**). In order to obtain rapid clinical remission of the extensive brain lesions, he was treated with mannitol (IV, 200 ml/6 hours), acyclovir (IV, 500 mg/8 hours) and high-dose methylprednisolone (IV, 1 g/day for five days). After 5 days at an oral dose of 80 mg/day, he was treated with oral prednisone at doses of 30 mg/day for three weeks. However, he did not report any improvement in clinical symptoms.

**Figure 4:**
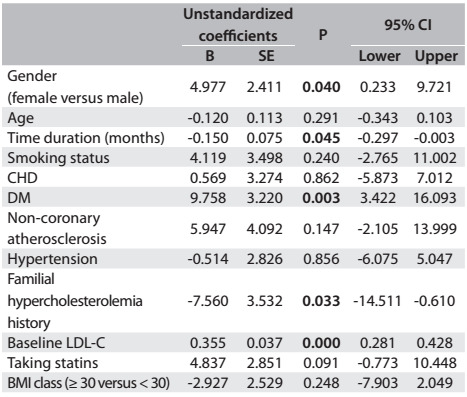
Magnetic resonance imaging of spinal cord, two months after admission, showing: hyperintense image with T2 weighting (A); and enhancement after gadolinium-diethylenetriamine pentaacetic acid (Gd-DTPA) administration (B).

At follow-up after two months, his paraparesis had increased in severity and extended to the left lower limb. The neurological examination revealed loss of sensation below T10 dermatome level and weakness grade 4/5 in the left lower limb. VEP examination was normal and CFS neuromyelitis optica (NMO)-IgG was negative, which is often helpful for ruling out the differential diagnoses of multiple sclerosis. In order to evaluate spinal cord lesions, it was decided to request a MRI scan. Spine MRI revealed diffuse abnormal signals that were hypointense on the T1-weighted images and hyperintense on the T2-weighted images, with heterogeneous enhancement after Gd-DTPA administration at T3/4 level (**Figure 4**).

**Figure 5:**
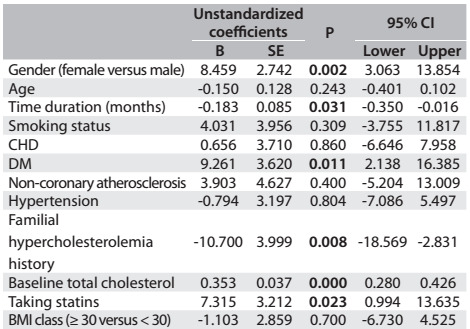
Magnetic resonance imaging of brain, three months after admission, showing: hyperintense image with T2 weighting (A); and enhancement after gadolinium-diethylenetriamine pentaacetic acid (Gd-DTPA) administration (B).

He received maintenance oral prednisone at doses of 30 mg/day. At follow-up after three months, he did not show any improvement from his previous symptoms and, moreover, these symptoms were accompanied by blurred vision and vomiting. A repeated brain MRI demonstrated an increased lesion in the right occipital lobe, which was hypointense on the T1 weighted images and hyperintense on the T2-weighted images and post-gadolinium T1-weighted image (**Figure 5**).

Administration of high-dose intravenous immunoglobulin (IVIG) was initiated (400 mg/kg/day for five days) and intravenous pulsed methylprednisolone, given as 1 g/day for five days and followed by a dose of 80 mg for three days. After these treatments, the headache and vomiting disappeared within a week and the other symptoms had improved remarkably. He was then treated with oral prednisone at doses of 60 mg/day for one week and 40 mg/day for two weeks. He was discharged home in good condition under treatment with prednisolone (30 mg/day).

## DISCUSSION

TIDD is a new independent clinical entity, intermediate between classic multiple sclerosis and acute demyelinating encephalomyelitis (ADEM). Despite decades of clinical and experimental research, the exact cause of TIDD remains unknown in most cases.

Occurrences of TIDD in the CNS are very rare. We conducted a systematic search in the main electronic databases (PubMed, Embase and Lilacs Library), to find papers that reported TIDD with brain and spinal cord involvement. In order to make the search as wide as possible, no limits were applied regarding the date of publication, the language used or the type of article (**Table 1**). There are fewer than 106 reported cases, and spinal TIDD is even rarer, with only 11 reported cases. Only two cases have involved both the brain and the spinal cord.^1^,^5^ The present report is on a young male with TIDD, in whom progressive lesions involved his brain and spinal cord.

**Table 1:** Systematic search of the literature performed on November 5, 2013

Electronic databases	Search strategy	Results	
		Found	Related
Medline (via PubMed)	"Inflammatory demyelinating disease" MeSH] AND "Brain" MeSH] AND "Spine" MeSH]	3	None of them demonstrated inflammatory demyelinating disease with brain and spinal cord involvement
Embase (via Elsevier)	"Inflammatory demyelinating disease" AND "Brain" AND "Spine"	106	2 reviews 2 case reports^1^,^5^
Lilacs	"Inflammatory demyelinating"	1	None of them demonstrated inflammatory demyelinating disease with brain and spinal cord involvement

MeSH = Medical Subject Headings; DeCS = Descritores em Ciências da Saúde.

TIDD lesions can mimic intracranial gliomas on conventional magnetic resonance images, may be difficult to diagnose and often result in surgical biopsy because of a suspected tumor.^6^–^8^ On images, TIDD and high-grade gliomas can both show contrast enhancement, perilesional edema, varying degrees of mass effect and central necrosis.^9^ Enhancement in TIDD cases is thought to correlate with the degree of macrophage infiltration and related blood-brain barrier breakdown, which is a relatively early histological finding in cases of acute lesions.^10^ Contrast enhancement also occurs in intracranial neoplasms, in high-grade gliomas in particular, because of blood-brain barrier breakdown in preexisting vessels and newly formed tumor capillaries that lack a blood-brain barrier. Therefore, lack of specificity of contrast enhancement renders it ineffective in differentiating TIDD from high-grade gliomas. MRS is useful for diagnosing demyelinating disease, monitoring its progression and evaluating the response to treatment.^11^ Unfortunately, the magnetic resonance spectroscopy (^1^H-MRS) findings revealed that large demyelinating lesions can show metabolic changes that are also found in malignant tumors. In the initial stage of the present case, because the lesion was a solitary dominant mass without typical white matter abnormalities of demyelination, with a clinical presentation suggestive of a space-occupying mass lesion, a diagnosis of intracranial neoplasm cannot easily be ruled out.

The differential diagnosis between TIDD and high-grade tumors can also be challenging using pathological evaluation. Five specific pathological patterns are common to TIDD and high-grade gliomas: hypercellularity, mitotic figures, pleomorphic reactive astrocytes with large, hyperchromatic nuclei, presence of necrotic areas, and areas of cystic degeneration or cavitation. Despite some similar histopathological features, TIDD and high-grade gliomas are different regarding vascularity. With regard to vascular changes, enlargement of endothelial cells and florid angiogenesis are distinctly absent in TIDD.^12^ Therefore, we suggest that stereotactic biopsy is necessary for confirming the correct diagnosis because of the progressive clinical signs, initial unresponsiveness to corticosteroid treatment and actively growing lesion.

TIDD patients tend to respond well to a course of intravenous steroids, and only a small percentage do not respond to this treatment or experience progression.^13^ IVIG is a treatment option for patients with severe demyelinating disease who have failed to respond to high-dose steroids. The present case demonstrated that long-term prednisolone and IVIG therapy may be beneficial, as has been reported in some cases of acute disseminated encephalomyelitis.^14^

## CONCLUSION

The present report describes an unusual case that was diagnosed as TIDD and showed progressive lesions involving the patient's brain and spinal cord. As demonstrated in our patient, TIDD lesions give rise to diagnostic dilemma for both the treating physician and the radiologist because of similarities with tumor lesions, regarding symptoms and imaging characteristics. A combination of strong clinical suspicion with characteristic MRI features and use of stereotactic biopsy may lead to correct diagnosing of demyelinating lesions. Long-term prednisolone and IVIG therapy is beneficial in treating TIDD.
